# Investigation on High-Temperature and High-Field Reliability of NMOS Devices Fabricated Using 28 nm Technology After Heavy-Ion Irradiation

**DOI:** 10.3390/mi16111216

**Published:** 2025-10-25

**Authors:** Yanrong Cao, Zhixian Zhang, Longtao Zhang, Miaofen Li, Shuo Su, Weiwei Zhang, Youli Xu, Dingqi Huang, Le Liu, Ling Lv, Xiaohua Ma

**Affiliations:** 1 School of Electronics & Mechanical Engineering, Xidian University, Xi’an 710071, China; 24041212505@stu.xidian.edu.cn (Z.Z.); 19041211991@stu.xidian.edu.cn (L.Z.); 24041212453@stu.xidian.edu.cn (M.L.); 23041212658@stu.xidian.edu.cn (S.S.); 23041212555@stu.xidian.edu.cn (W.Z.); 25041212657@stu.xidian.edu.cn (Y.X.); 25041212697@stu.xidian.edu.cn (D.H.); 25041212647@stu.xidian.edu.cn (L.L.); 2 State Key Discipline Laboratory of Wide Bandgap Semiconductor Technology, School of Microelectronics, Xidian University, Xi’an 710071, China; llv@xidian.edu.cn (L.L.); xhma@xidian.edu.cn (X.M.)

**Keywords:** heavy ion irradiation, high-temperature, high-field, degradation

## Abstract

This paper investigates the degradation of 28 nm technology NMOS devices under high-temperature and high-field conditions following heavy-ion irradiation. The effects of stress time, stress magnitude, temperature, device structural dimensions, and heavy-ion radiation fluence on device degradation were analyzed. The results indicate that under positive gate bias stress, the threshold voltage of NMOS devices exhibits a continuous positive shift. Increased stress time, higher stress magnitude, elevated temperature, and reduced device structural dimensions all aggravate device degradation. The combined effects of electrical stress and radiation lead to a degradation that initially decreases and then increases. This is because the trap charges generated in the gate oxide layer by radiation are positive charges at low fluence, compensating for the negative charges generated under electrical stress, thereby reducing degradation. However, at high fluence, the negative interface trap charges increase, while radiation also generates positive charges in the shallow trench isolation (STI) region. These two factors collectively contribute to increased device degradation.

## 1. Introduction

With humanity’s deep space exploration advancing progressively into farther and unknown regions of the universe, spacecraft impose increasingly stringent requirements on the performance and reliability of their onboard electronic devices [1,2,3,4]. To this day, CMOS technology remains the dominant platform in fields such as healthcare, engineering applications, and aerospace electronics, while radiation effects in NMOS devices represent a critical cause of logic errors and failures in CMOS integrated circuits [5,6]. In fact, as critical components of aerospace electronic systems, such devices face reliability challenges beyond those mentioned—they are susceptible not only to performance degradation due to radiation exposure but also to performance degradation or complete failure under prolonged high-temperature and high-field (HT/HF) operating conditions. These complex failure mechanisms cause significant challenges to the radiation hardening of aerospace electronics.

Furthermore, as feature sizes continue to shrink to the nanoscale following Moore’s Law, conventional SiO_2_-based devices are confronted with several issues, such as increased gate leakage current, enhanced quantum tunneling effects, and reduced gate oxide breakdown voltage [7,8]. Although the emergence of high dielectric constant materials has alleviated these challenges, the poor interface properties between high-k dielectric and silicon necessitate the incorporation of a thin SiO_2_ interlayer between the high-k dielectric and the Si substrate, resulting in a multi-interfacial structure [9,10]. While this structure provides advantages such as low power consumption, high integration density, and fast switching speed, the higher trap density in the high-k layer and the dual-interface structure also complicate the degradation mechanisms under temperature bias stress and radiation exposure [11].

In the field of radiation effects, heavy ions present in the space radiation environment, which possess high Linear Energy Transfer (LET) values, have a significant impact on microelectronic devices. The existing research indicates that heavy ion irradiation poses a major threat to the space application of high-k HfO_2_ MOS devices. Choi et al. found that as the gate oxide thickness decreases and the ion LET and irradiation fluence increases, the degradation caused by Radiation-Induced Soft Breakdown (RSB) becomes more severe [12]. RSB can lead to a gate current of up to 1 μA, correspondingly increasing the device power consumption to 1 μW [13], which has an impact on low-power aerospace microelectronic systems. Conley et al. investigated the effects of irradiation on ultrathin oxide films using ^127^I ions with an LET value of 60 MeV·cm^2^/mg [14]. Their study revealed that heavy ion irradiation can degrade device reliability even when the device is powered off. Regarding reliability under high-temperature and high-field conditions, Mukhopadhyay et al. conducted a study on Positive Bias Temperature Instability (PBTI) in NMOS devices with high-k dielectrics. Their findings indicated that interface traps generated during PBTI dominate the threshold voltage degradation, while contributions from oxide traps and trapped electrons are relatively minor [15]. Chen D. Y. et al. examined the impact of PBTI stress on the 1/f noise characteristics of FinFETs, exploring the distribution of trap density within the gate dielectric layer under stress. They observed that defects are primarily localized in the high-k layer and that short-channel devices exhibit more significant degradation under PBTI stress [16]. Regarding studies on the coupling of irradiation effects with high-temperature and high-field conditions, Bagatin et al. investigated the impact of Bias Temperature Instability (BTI) on the Single Event Upset (SEU) rate in nanoscale devices and circuits. Their results indicate that when the parameter variation induced by BTI is limited to within 10%, the SEU rate does not exhibit significant changes [17]. Harada et al. examined the Single Event Effects (SEE) in nanoscale CMOS devices under BTI stress, revealing that the Single Event Transient (SET) pulse width exhibits weak temperature dependence but is more sensitive to static and dynamic electrical stress. Moreover, the SET pulse width increases with intensified electrical stress [18]. Wang Zhen et al. developed an analytical model for the SET pulse width in nanoscale MOSFETs under high-temperature and high-field conditions [19]. However, research combining the effects of heavy-ion irradiation with the high-temperature and high-field reliability of small-scale high-k MOS devices remains relatively scarce, unsystematic, and insufficiently in-depth.

Therefore, this study focuses on NMOS devices with high-k dielectrics fabricated using 28 nm technology. The devices were irradiated with heavy ions (^181^Ta^35+^), followed by high-temperature and high-field experiments under various conditions. Key characteristic parameters were extracted to analyze the degradation patterns. The findings aim to provide an evaluation standard for enhancing the reliability of small-sized aerospace electronic devices.

## 2. Experimental Devices and Conditions

**Figure 1:**
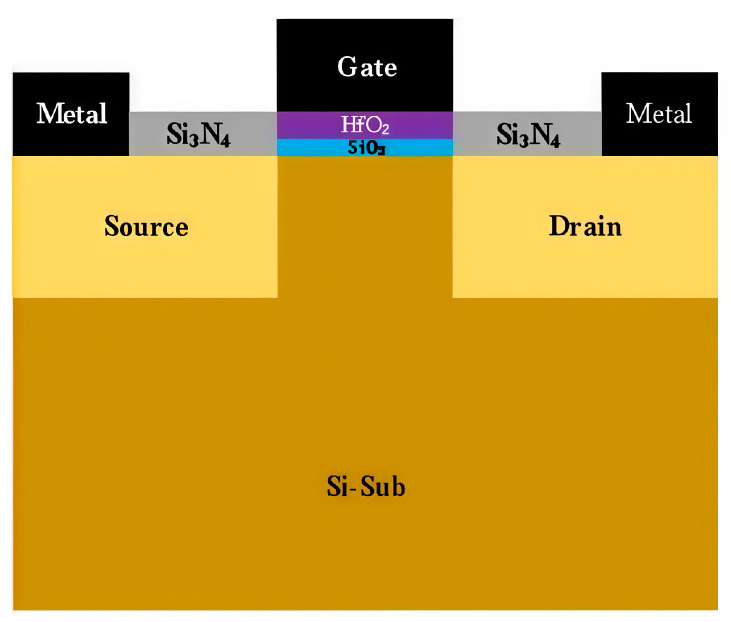
A 28 nm process NMOS device structure.

The devices used in the experiment were provided by Beijing Microelectronics Technology Institute, Beijing, China, and were fabricated using a standard 28 nm MOSFET process. The gate dielectric consisted of an HfO_2_/SiO_2_ high-k dielectric bilayer dielectric stack. Figure 1 shows the schematic of the device. The devices used in the experiment operate at two voltage levels: 0.9 V and 3.3 V. The equivalent gate oxide thicknesses for these devices are 1.43 nm and 6.19 nm.

In the experiment, the electrical stress and testing equipment adopted the PDA semiconductor device testing system. The heating device utilized the precision constant temperature workbench WT-3000-12S. The radiation device was the Heavy Ion Research Facility in Lanzhou, China (HIRFL). The irradiation source used in this work was ^181^Ta^35+^, with an ion flux set to 10^5^ ions/(cm^2^·s). The total fluence was controlled at 7 × 10^6^ ions/cm^2^, 1 × 10^8^ ions/cm^2^, and 1 × 10^10^ ions/cm^2^, respectively. Detailed parameters of the irradiation source are summarized in Table 1.

**Figure 2:**
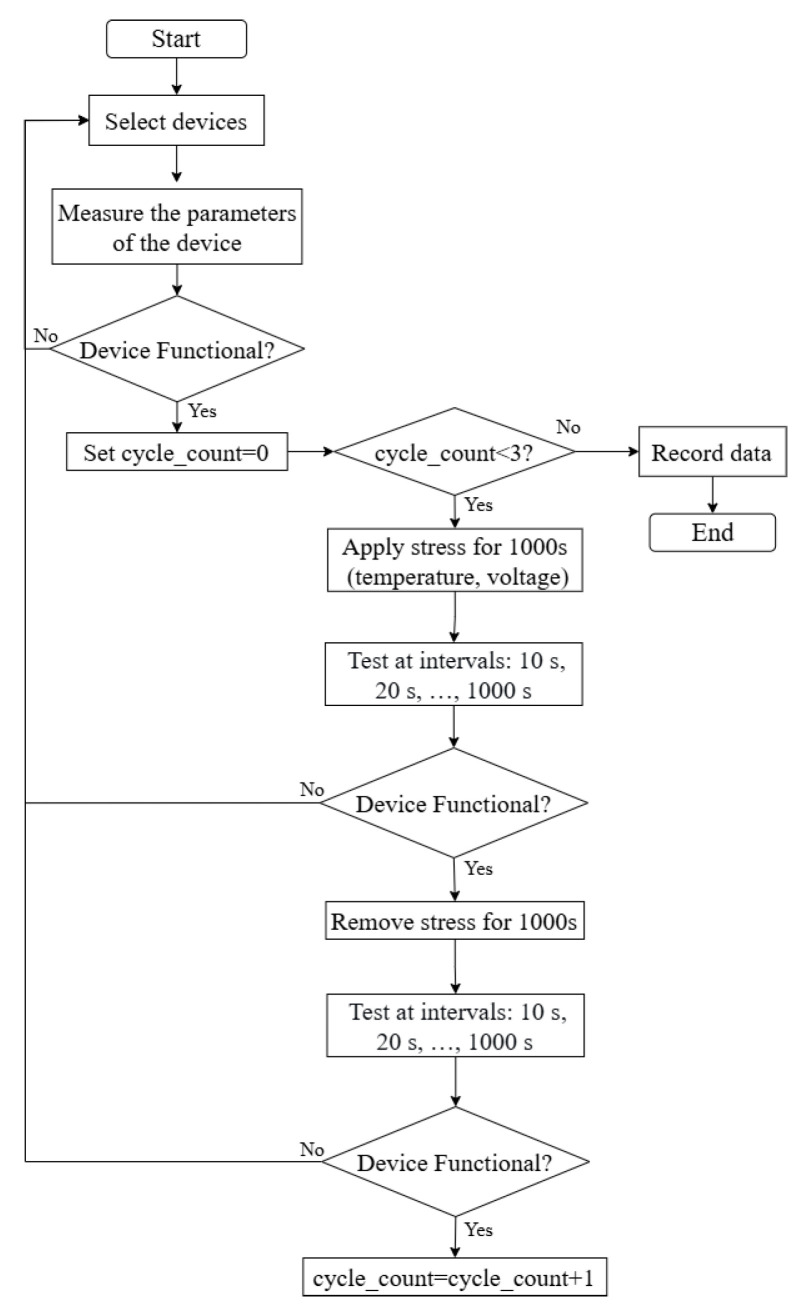
Testing procedure for the experiment.

The experiment investigated the high-temperature and high-field degradation under various post-irradiation conditions by systematically varying parameters such as electrical stress and device dimensions, as summarized in Table 2. The detailed experimental testing procedure is illustrated in Figure 2.

In this experiment, three consecutive stress cycles were implemented. Each cycle was defined as 1000 s of applied stress followed by 1000 s of stress removal. Accordingly, the time intervals of 0–2000 s, 2000–4000 s, and 4000–6000 s correspond to Cycle 1, Cycle 2, and Cycle 3, respectively.

## 3. Experimental Results and Analysis

### 3.1. Effect of Electrical Stress Time on Device Degradation

**Figure 3:**
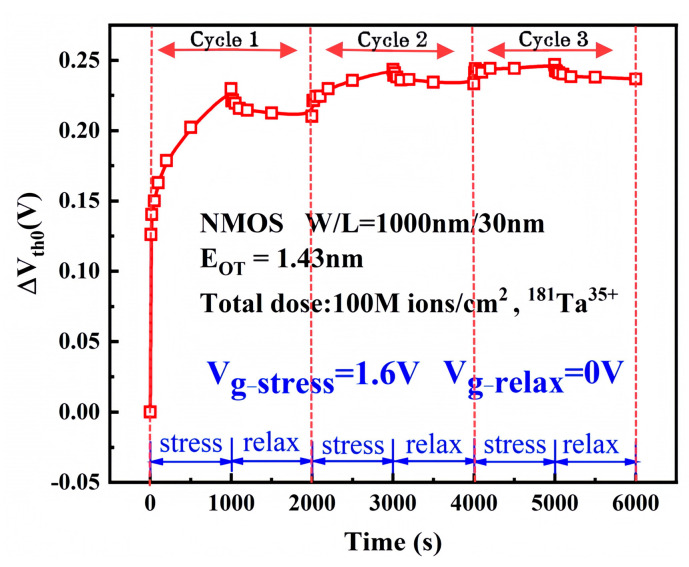
Variation of $\Delta V_{th0}$ with stress time.

The NMOS devices used in this experiment feature a gate width-to-length ratio of 1000 nm/30 nm. A constant gate stress voltage of 1.6 V was applied. The heavy-ion fluence for the group reached 1 × 10^8^ ions/cm^2^. The initial threshold voltage at time zero is denoted as V_th0_. The initial threshold voltage was 0.29 V. The degradation of the threshold voltage ($\Delta V_{th0}$) as a function of electrical stress time, extracted at various time points, is shown in Figure 3.

After irradiation, the threshold voltage (V_th_) of the NMOS devices exhibits a positive shift with increasing electrical stress duration, indicating progressive device degradation. The majority of this degradation occurs during the initial stress cycle and demonstrates gradual saturation with prolonged stress time.

The $\Delta V_{th0}$ variation primarily arises from newly generated interface traps, oxide traps under electrical stress, and native defects in the dielectric layer capturing trapped electrons. This functional relationship can be expressed as [20]:

(1) $$\Delta V_{th0}=\Delta V_{ot}+\Delta V_{it}+\Delta V_{et}=\frac{-qN_{ot}}{C_{ox}}+\frac{-qN_{it}}{C_{ox}}+\frac{-qN_{et}}{C_{ox}}$$

**Figure 4:**
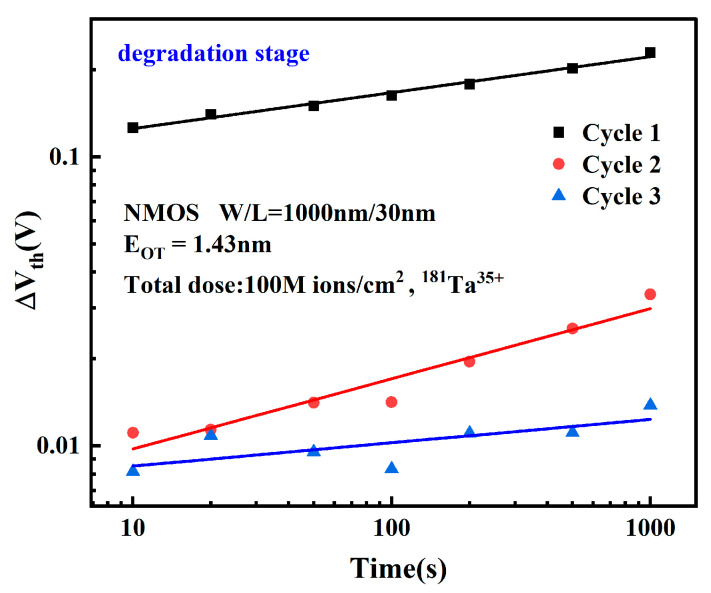
Relationship between the change in threshold voltage during each stress application phase per cycle and time in a double-logarithmic coordinate system.

$N_{ot}$ is the net trap charge density of the oxide layer, $N_{it}$ is the net trap charge density of the double interface, $N_{et}$ is the trapped electron density, and $C_{ox}$ is the oxide layer capacitance. In order to study the relationship between degradation and stress time more clearly, the stress degradation magnitudes of the three cycles were plotted in logarithmic coordinates. The $\Delta V_{\mathrm{th}}$ was calculated with reference to the initial threshold voltage at the beginning of each 1000 s cycle, as shown in Figure 4.

The ratio of threshold voltage shift to its initial value exhibits an approximately linear relationship with electrical stress time in log-log coordinates, conforming to the expression:

(2) $$\mathrm{lg}(\Delta V_{\mathrm{th}})\propto A_{0}+n\;\mathrm{lg}\;t$$

Transformed to linear coordinates, it satisfies:

(3) $$\Delta V_{th}\propto A_{1}t^{n}$$

where *n* is the time degradation exponent and *A*_1_ is the time-dependent fitting coefficient. During the electrical stress phase, the degradation approaches saturation as the number of stress cycles increases.

**Figure 5:**
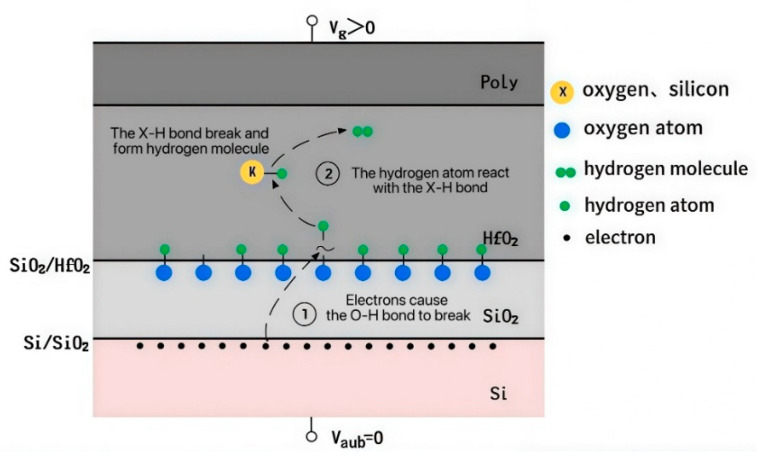
R-D process of NMOS under electrical stress.

These phenomena are explained by a modified Reaction-Diffusion (R-D) model [21]. As illustrated in Figure 5: Under positive bias temperature stress applied to the NMOS device, the device operates in strong inversion. Some electrons in the channel can tunnel into the gate dielectric layer. During their transport across the Si/SiO_2_ and SiO_2_/HfO_2_ interfaces, these electrons trigger reactions that break O–H and Si–H bonds formed due to the introduction of H elements during the manufacturing process, thereby generating Si- or O-interface traps and releasing hydrogen atoms (Process ① in Figure 5). The released hydrogen atoms subsequently diffuse into the HfO_2_ layer, where they further react to form X-defects and hydrogen molecules (Process ② in Figure 5). Increasing stress time elevates broken bond density, interface trap density, and hydrogen atom diffusion into HfO_2_, thereby amplifying the V_th_ positive shift. Concurrently, channel electrons captured by oxygen vacancies convert neutral oxygen vacancies into negatively charged centers, further contributing to positive V_th_ shift. However, prolonged stress reduces both available bonds for scission and diffusion-driving concentration gradients, collectively causing progressive degradation rate reduction and eventual saturation.

**Figure 6:**
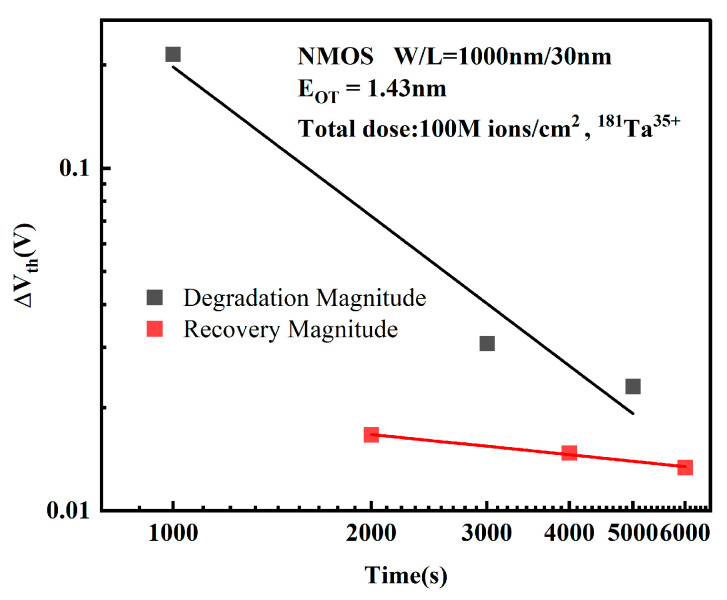
Degradation and recovery magnitudes per cycle in logarithmic coordinates.

Figure 6 depicts the degradation and recovery magnitudes over three stress cycles in log-log coordinates. The degradation magnitude is defined as the difference between the threshold voltage at a specified time point during the 1000 s stress phase of a cycle and the initial threshold voltage at the beginning of that stress phase. The recovery magnitude is defined as the difference between the threshold voltage at a specified time point during the 1000 s stress-removal phase of a cycle and the initial threshold voltage at the beginning of that stress-removal phase.

**Figure 7:**
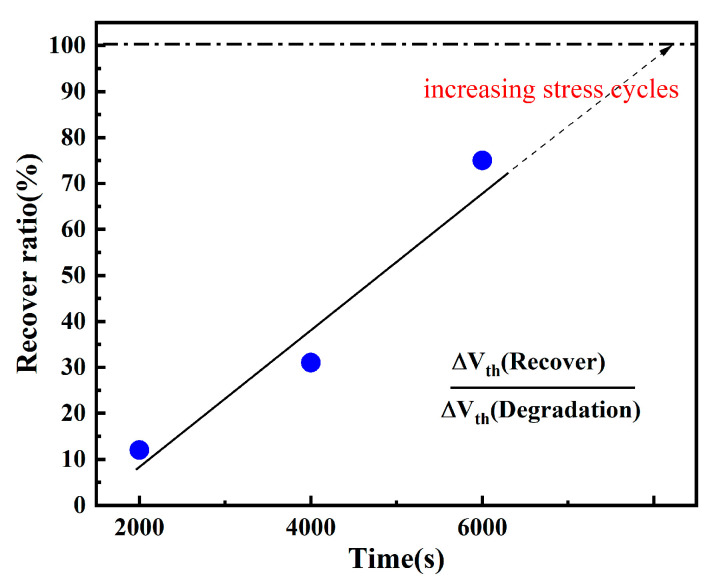
The recovery rate of the device changes with the number of cycles.

The recovery ratio extracted during each stress cycle is shown in Figure 7. The recovery ratio is defined as the percentage of the recovery magnitude relative to the degradation magnitude within the same stress cycle [22]. Recovery primarily occurs due to hydrogen substance (generated during the stress phase) back-diffusing to the interface and re-passivating interfacial bonds. However, since the quantity of generated hydrogen substance is limited and only a fraction successfully back-diffuses to the interface, the recovery magnitude remains smaller than the degradation magnitude. Studies indicate that device recovery is incomplete and eventually reaches an asymptotic saturation state. Furthermore, the final degradation level is closely correlated with the recovery stress magnitude, stress duration, stress temperature, and stress cycle period [23].

### 3.2. Effect of Electric Stress on Device Degradation

**Figure 8:**
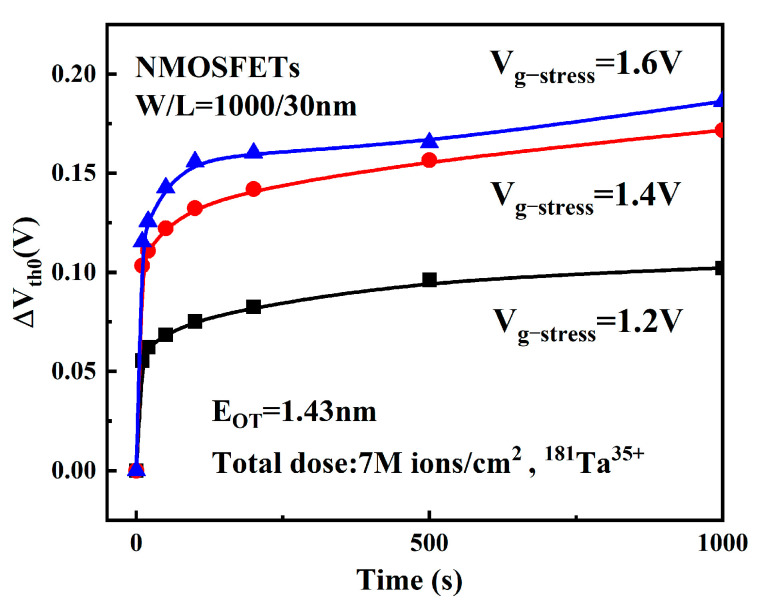
Degradation of V_th_ under different V_g−stress_ after radiation.

Figure 8 depicts the V_th_ degradation over time under different stress conditions post-irradiation. As shown in the figure, the V_th_ of NMOS devices exhibits a positive shift with increasing stress time across all three stress conditions. Furthermore, the magnitude of V_th_ degradation ($\Delta V_{th0}$) increases progressively with higher gate stress voltage (V_g−stress_).

**Figure 9:**
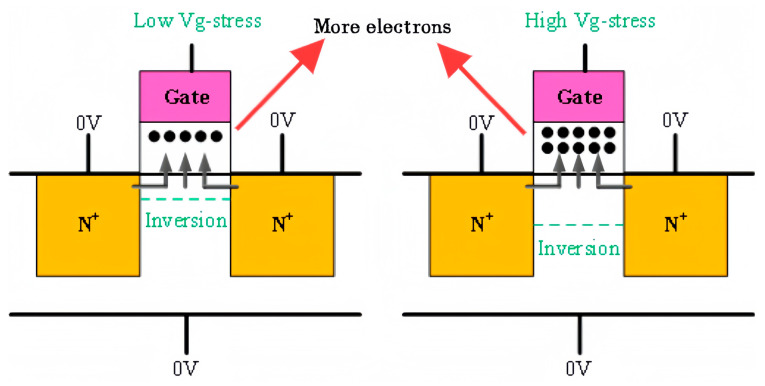
Degradation mechanisms of device characteristics under different voltages.

Under high electrical stress, the inversion layer widens and the electron concentration increases, enabling a greater number of electrons to enter the gate oxide layer, thereby exacerbating device degradation [24]. Figure 9 shows the mechanism of device degradation under high stress. Furthermore, when the thickness of the gate dielectric layer is constant, the increase in electric stress will lead to an increase in the longitudinal electric field intensity of the oxide layer. The electric field enhancement makes it easier for the electrons in the channel to tunnel to the interface and participate in the interface reaction and makes the interface bond more likely to break and produce hydrogen substance. These produced hydrogen substances then drift more rapidly under the electric field into the HfO_2_ layer, reacting to produce more oxide layer traps. As a result, the degradation of NMOS devices under high-temperature and high-field conditions becomes more pronounced.

### 3.3. Effect of Temperature Stress on Device Degradation

**Figure 10:**
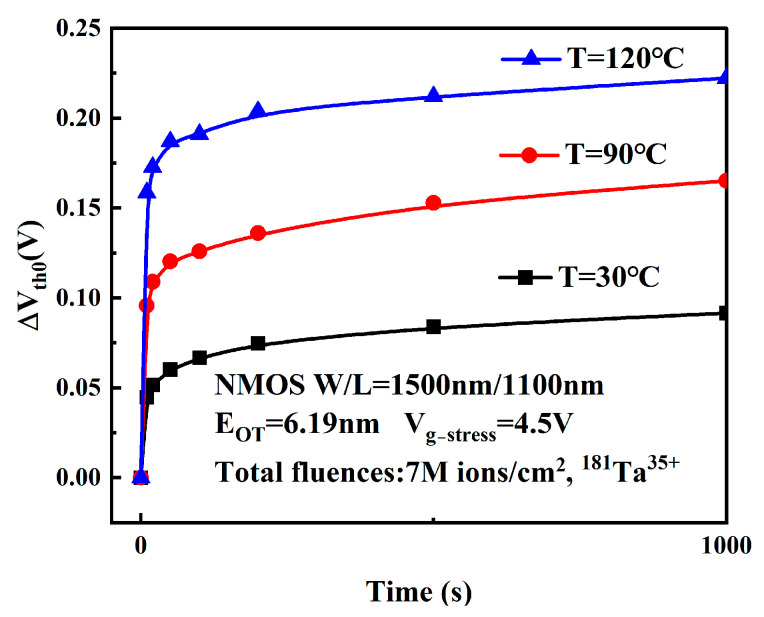
Variation of $\Delta V_{th0}$ with time under different temperature stresses.

Figure 10 displays the degradation magnitude of the V_th_ over time under different temperature stresses after radiation exposure. As can be observed from Figure 10, higher stress temperatures result in more pronounced degradation of the threshold voltage.

This phenomenon occurs due to the following reasons: elevated temperature accelerates the scission of passivated interfacial bonds (Si–H and O–H bonds), and higher temperature increases the diffusion rate of hydrogen substance (generated during the R-D process) into the gate oxide bulk. Analysis of the V_th0_ evolution reveals that $\Delta V_{th0}$ obeys the following relationship with temperature stress [23]:

(4) $$\Delta V_{th0}\propto C\exp(-\frac{E_{a}}{kT})$$

**Figure 11:**
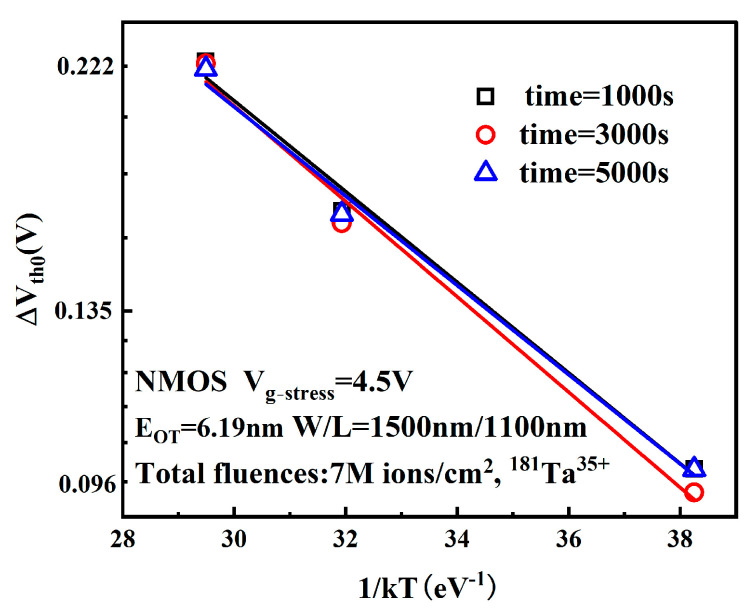
$\Delta V_{th0}$ as a function of 1/kT.

This empirical formula is the Arrhenius Equation, where C is the proportionality coefficient, $E_{a}$ denotes activation energy, *k* represents the Boltzmann constant, and T is absolute temperature. Figure 11 displays linear fitting trends of threshold voltage degradation versus 1/kT in semi-logarithmic coordinates at electrical stress times of 1000 s, 3000 s, and 5000 s. The slope of the fitted line corresponds to the activation energy, which quantifies the difficulty of device degradation under the given temperature stress conditions [25].

Since $E_{a}$ remains constant for identical devices, the three curves exhibit nearly identical slopes, yielding $E_{a}$ ≈ 0.09 eV. This value is lower than most reported $E_{a}$ values in the literature, indicating that small-scale MOSFETs are more susceptible to degradation under high-temperature and high-field conditions following heavy-ion irradiation [26].

### 3.4. Effect of Gate Length on Device Degradation

**Figure 12:**
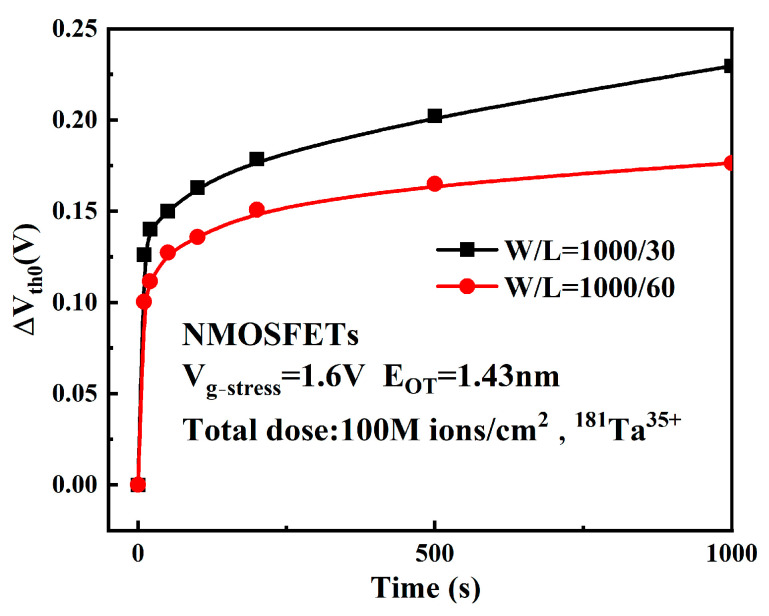
$\Delta V_{th0}$ of devices with different gate lengths.

In order to explore the effect of the gate length on the device, two devices with the gate lengths of 30 nm and 60 nm were selected for experiments, and the changes in the threshold voltage of the device over time were obtained as shown in Figure 12.

**Figure 13:**
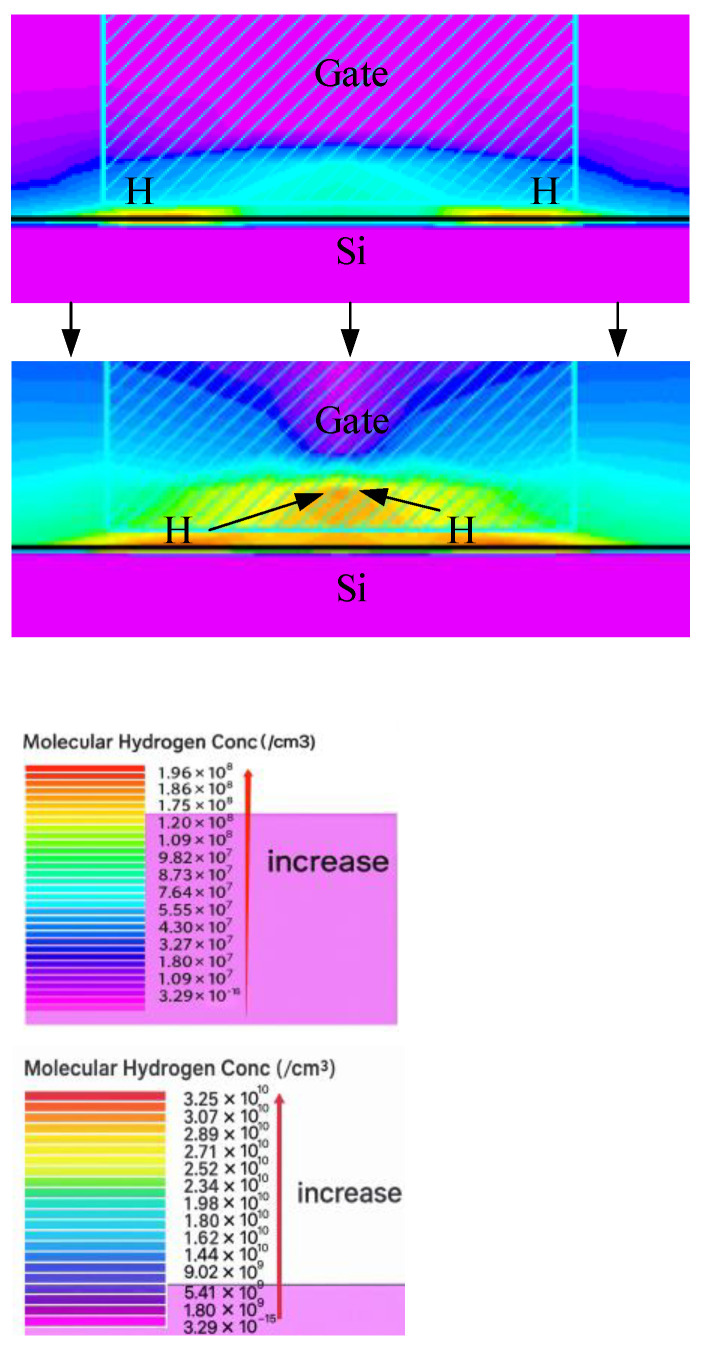
Simulation diagram of H atom generation and diffusion.

As shown in the figure, reduced gate length L in post-irradiation devices leads to aggravated degradation. In the process of applying electrical stress, the degree of damage along the length of the channel is not the same, which may be caused by the uneven distribution of potential defects at the gate center, the gate-drain, and the gate-source junction, and the manufacturing process of the device will introduce more potential defects at the edge of the gate oxide layer [27]. A two-dimensional (2D) simulation of post-irradiation bias instability in the device was performed using Silvaco TCAD software (Atlas 5.34.0.R) under an electrical stress of 1.6 V. Potential damage defects will make the R-D reaction first occur on both sides of the gate, as shown in Figure 13. Simulation results show that H atoms generated on both sides of the gate indicate more interface trap charges at the edge of the gate, indicating that the degradation degree at the edge of the gate is greater than that at the center of the gate.

As the channel length L decreases, the ratio of ∆L/L between the length of the serious degradation zone on both sides of the channel and the total channel length increases, and the edge effect has a greater influence on the device. Therefore, the reduction in the gate length of the device makes the device show more serious degradation.

### 3.5. Effect of Gate Width on Device Degradation

**Figure 14:**
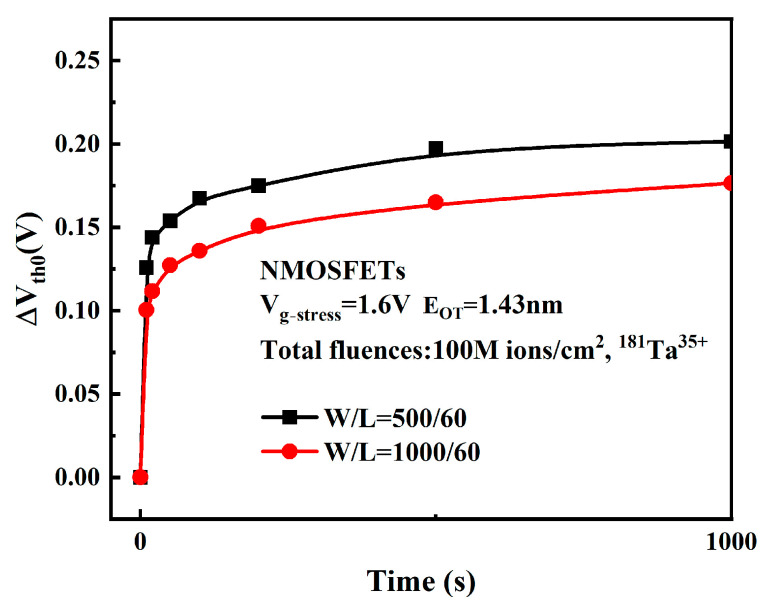
$\Delta V_{th0}$ of devices with different gate widths.

As with the gate length, we selected two devices with gate widths of 500 nm and 1000 nm, respectively, to obtain the high-temperature and high-field degradation effect of the device, and the change in the threshold voltage of the device with time is shown in Figure 14.

**Figure 15:**
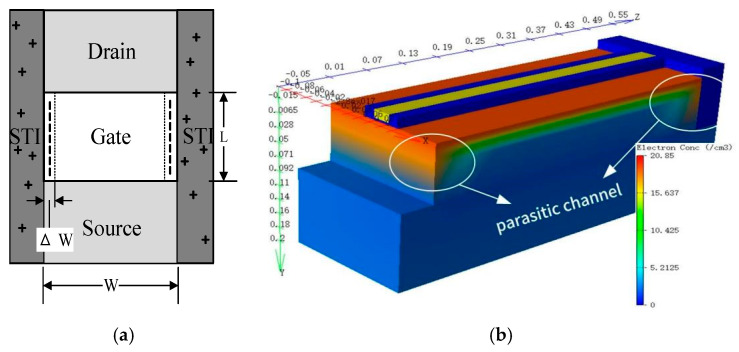
(**a**) Parasitic channel near STI interface structure diagram. (**b**) Parasitic channel near STI interface simulation diagram.

As evidenced in the figure, reduced gate width (W) leads to more pronounced device degradation. Studies show that mechanical stress is generated during STI structure formation, and mechanical stress can lead to higher density interface trap charge at the interface between STI and gate [28,29]. For NMOS, the interface trap with negative charge will lead to positive drift of device threshold voltage. The interface trap effect caused by mechanical stress of STI increases with the decrease in gate width W, which makes the degradation of devices with smaller gate width more serious. In addition, a three-dimensional (3D) simulation of post-irradiation bias instability in the device was performed using Silvaco TCAD under an electrical stress of 1.6 V. As shown in Figure 15, a large number of trap positive charges are introduced into STI during the radiation process, and parasitic channels will be generated near the STI interface [11]. In the simulation figure, it can be obviously observed that electron concentration near STI on both sides is higher than other positions. The leakage current at the edge of STI will lead to degradation of device characteristics. With the shortening of the MOS gate width, the degradation ratio ∆W/W caused by the parasitic effect of STI increases, so that devices with a small gate width have greater degradation of device characteristics.

### 3.6. Effect of Heavy Ion Fluence on Device Degradation

**Figure 16:**
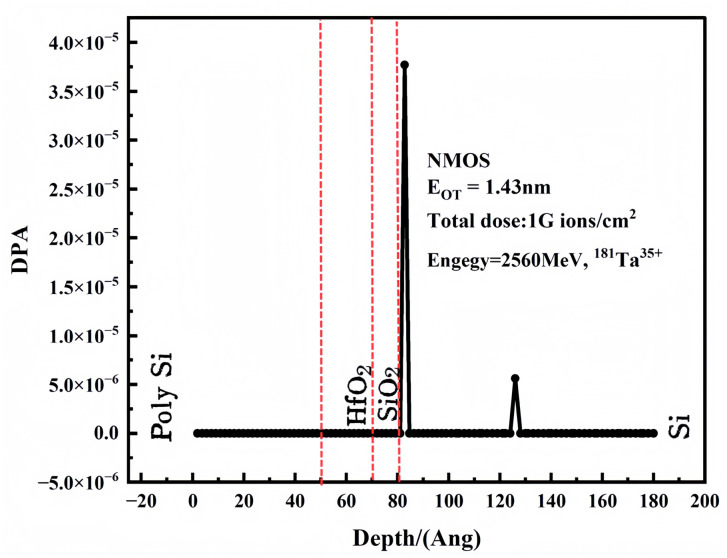
Distribution of dpa calculated for 2560 MeV ^181^Ta^35+^ in Kinchin-Pease (K-P) mode.

Different irradiation conditions induce varying degrees of damage within the device. To investigate the atomic displacements caused by Ta ion incidence in the insulating layer, this section employs SRIM-2013 (The Stopping and Range of Ions in Matter) to calculate the irradiation damage in the target material. The damage degree is quantified using the dpa (displacements per atom) metric, which represents the average number of displacements per atom under a given fluence. As shown in Figure 16, the atomic displacement within the insulating layer is negligible under the experimental irradiation dose.

**Figure 17:**
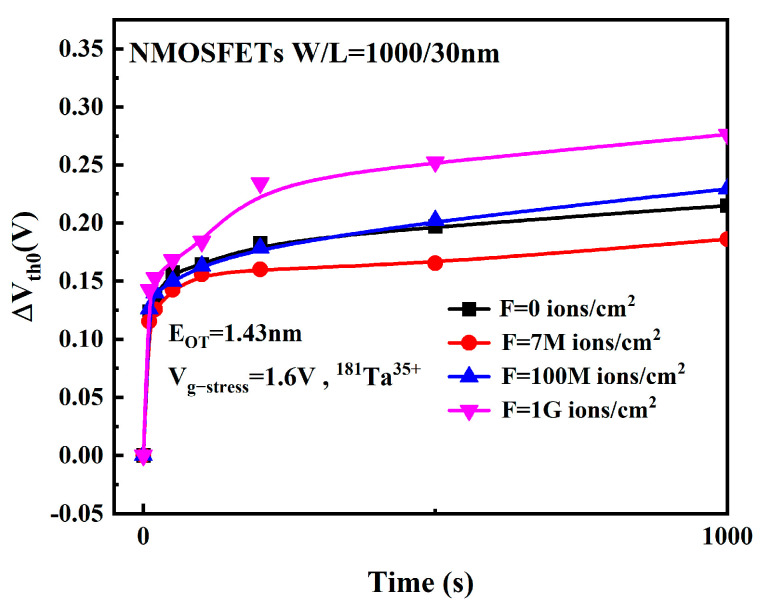
Variation of $\Delta V_{th0}$ with time under different heavy ion fluences.

Figure 17 depicts the $\Delta V_{th0}$ as a function of electrical stress time under varying heavy-ion fluence conditions. As observed, the V_th_ exhibits a consistent positive shift with increasing stress time across all fluence levels. Notably, the degradation magnitude decreases with elevated fluence prior to reaching 7 × 10^6^ ions/cm^2^, but increases monotonically beyond this critical fluence threshold.

**Figure 18:**
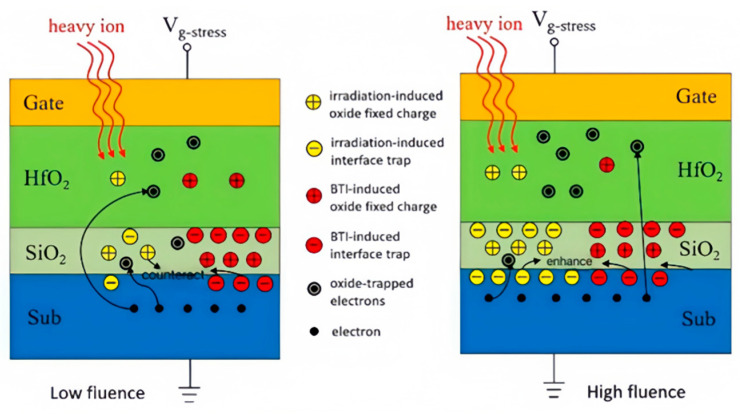
Influence of radiation on device degradation at different injection rates.

The main reason is that the interface trap and oxide layer trap generated by radiation are related to the amount of heavy ion radiation. The rate of interface trap charge generation is slower than that of oxide layer trap charge, and it requires a certain amount of radiation and time to generate interface trap charge. Meanwhile, interface trap charge can exist for a long time. As shown in Figure 18, radiation generates two kinds of charges in the double-interface oxide layer: the oxide layer trap charge and the interface trap charge. At low fluence levels, the number of positive oxide traps is more than the number of interface traps, so that the negative interface charge generated under electrical stress is offset, and the threshold voltage drift of the device is smaller than that of the unirradiated device.

Meanwhile, for small-sized MOS devices, heavy ion radiation generates a large number of positive charges in the STI region, which gradually plays a major role in the degradation of device characteristics. On the one hand, the proportion of positive charge that can be compensated by coupling from the electrons entering the oxide layer from the channel decreases, and at the same time, the radiation introduces more negative interface trap charges under the large amount of injection. These negative interface charges strengthen the negative interface charges introduced by the electrical stress, making the threshold positive drift more severe. Secondly, the positive trap charges captured by STI have no electronic compensation effect under high fluence, which increases the concentration of electrons in the STI parasitic channel and generates more electrons into the gate oxide layer under the action of electrical stress, resulting in more serious device degradation.

## 4. Conclusions

In this paper, the effects of different factors on device characteristics are studied based on high-temperature and high-field experiments after radiation. As the electrical stress time increases, the threshold voltage of the MOSFET exhibits a progressive positive shift. This behavior indicates that the stress-generated interface and oxide traps are negative charges. Furthermore, the threshold voltage degradation follows a power-law relationship with the stress time. Increasing the electrical stress increases the electric field intensity at the channel, which accelerates the electrons in the channel to enter the interface more easily to participate in the R-D reaction and finally makes the degradation degree of the device more serious. Investigation on the correlation between device structural dimensions and degradation indicates that the edge effect makes the degradation increase with the decrease in gate length. The parasitic channel formed by heavy ion radiation near the STI interface increases the threshold voltage of devices, and the existence of the parasitic channel leads to more serious degradation of devices with small gate width. The degradation of devices decreases at first and then increases due to the joint action of electrical stress and radiation. At low fluence, more positive oxidation layer trap charges are mainly generated, which offset the negative trap charges generated by electrical stress and reduce the degradation of devices. At high fluence, a large number of negative interface charges and STI trap positive charges are generated, the combination of these two with the negative trap charge generated by the electrical stress will increase the degradation of the device.

## Figures and Tables

**Table 1:** Parameters of heavy ion radiation source.

Parameter	Value
Radiation ions	^181^Ta^35+^
Energy/MeV	2560
LET/MeV/(mg/cm^2^)	70.06
Range/μm	149.22

**Table 2:** Experimental parameters.

No.	Influence Factors	Constant Parameters	Varied Parameters
1	Electrical stress	T = 30 °C	V_g−stress_ = 1.2 V, 1.4 V, 1.6 V
2	Temperature	V_g−stress_ = 4.5 V	T = 30 °C, 90 °C, 120 °C
3	Gate length	V_g−stress_ = 1.6 V, T = 30 °C, W = 1000 nm	L = 30 nm, 60 nm
4	Gate width	V_g−stress_ = 1.6 V, T = 30 °C, L = 30 nm	W = 1000 nm, 500 nm
5	Heavy ion fluence	V_g−stress_ = 1.6 V, T = 30 °C	Heavy Ion Fluence = 0 ions/cm^2^, 7 × 10^6^ ions/cm^2^, 1 × 10^8^ ions/cm^2^, 1 × 10^10^ ions/cm^2^

## Data Availability

The data presented in this study are available on request from the corresponding author.
